# A Divided House in a Cathedral City

**Published:** 1887-09-24

**Authors:** 


### A DIVIDED HOUSE IN A CATHEDRAL CITY.

We publish the following from a correspondent, in the interests of justice. No doubt there is another side to the question, to which for the same reason we shall be glad to give the fullest publicity :—

"Can you spare me space in your columns for a few words? I take great interest in a small hospital in one of our cathedral towns. The hospital itself is a pleasant one, situated within easy distance of the river and the country. The salaries received by the nurses upon the staff are as good as are usually given in provincial hospitals; in fact, everything outwardly appears desirable, and yet a spirit of discontent is rife within the place. I often hear the question asked, 'Why are the nurses not happy, and why are there such constant changes?' There is but one reply possible: 'While the matron and house-surgeon are at variance there can be no peace throughout the little kingdom which they govern.' In this case, one up till now has always been the aggressor, and the other has borne patiently the insinuations and innuendos launched against him in public and in private. Personal acquaintance has proved the falsity of many of these malicious reports, which could have sprung from but one source.

"Matters grow daily worse, till now, a nurse, when entering on her duties, is told by the matron that the house is divided, and the new-comer must be on one side or the other. What does this mean? In plain language, evince a friendly feeling towards the wrong side, and you may vacate—to find another post as best you can—minus a matron's testimonial. This, as any experienced nurse knows, is equivalent to telling a professional nurse to throw up her means of earning a livelihood. Who in these days of strong competition would accept a nurse without a testimonial from her former matron?

"The difficult part of the matter is, that nearly all are in favour of the house-surgeon, who is thorough and conscientious in his work, uniformly civil to the nurses, and generally popular throughout the hospital; yet, for reasons stated above, none dare sail under their true colours. This miserable state of things must unfortunately continue. Neither matron nor house-surgeon is likely to leave; open war becomes imminent; and the hospital, once so peaceful, is getting up its name as a hotbed of strife, and so uncomfortable that constant change is the result.

"Can anyone, through the columns of The Hospital, suggest a course of action to be pursued by the nursing staff, a course which will allow of straight sailing, yet not necessitate toadying? Bearing in mind always that the matron is 'the powers that be,' as far as the nurses are concerned, and she ordains that one side or the other be taken in this civil war. The house-surgeon says nothing and asks for nothing; he merely does his duty. This simple plan of action speaks for itself, and his partisans are many. Still, his fellow-workers dare not show themselves as such, and I, as an outsider, and a friend to fair dealing and peace, feel sorry that our hospital should be the scene of dissension, and in many cases of deceit."

